# Investigation of Antifungal Mechanisms of Thymol in the Human Fungal Pathogen, *Cryptococcus neoformans*

**DOI:** 10.3390/molecules26113476

**Published:** 2021-06-07

**Authors:** Kwang-Woo Jung, Moon-Soo Chung, Hyoung-Woo Bai, Byung-Yeoup Chung, Sungbeom Lee

**Affiliations:** 1 Radiation Research Division, Advanced Radiation Technology Institute, Korea Atomic Energy Research Institute, Jeongeup-si 56212, Jeollabuk-do, Korea; mschung@kaeri.re.kr (M.-S.C.); hbai@kaeri.re.kr (H.-W.B.); bychung@kaeri.re.kr (B.-Y.C.); 2 Department of Radiation Science and Technology, University of Science and Technology, Daejeon 34113, Yuseong-gu, Korea

**Keywords:** thymol, *Cryptococcus neoformans*, antifungal activity, calcium homeostasis, *N*-glycosylation, ergosterol

## Abstract

Due to lifespan extension and changes in global climate, the increase in mycoses caused by primary and opportunistic fungal pathogens is now a global concern. Despite increasing attention, limited options are available for the treatment of systematic and invasive mycoses, owing to the evolutionary similarity between humans and fungi. Although plants produce a diversity of chemicals to protect themselves from pathogens, the molecular targets and modes of action of these plant-derived chemicals have not been well characterized. Using a reverse genetics approach, the present study revealed that thymol, a monoterpene alcohol from *Thymus vulgaris* L., (Lamiaceae), exhibits antifungal activity against *Cryptococcus neoformans* by regulating multiple signaling pathways including calcineurin, unfolded protein response, and HOG (high-osmolarity glycerol) MAPK (mitogen-activated protein kinase) pathways. Thymol treatment reduced the intracellular concentration of Ca^2+^ by controlling the expression levels of calcium transporter genes in a calcineurin-dependent manner. We demonstrated that thymol decreased *N*-glycosylation by regulating the expression levels of genes involved in glycan-mediated post-translational modifications. Furthermore, thymol treatment reduced endogenous ergosterol content by decreasing the expression of ergosterol biosynthesis genes in a HOG MAPK pathway-dependent manner. Collectively, this study sheds light on the antifungal mechanisms of thymol against *C. neoformans*.

## 1. Introduction

The incidence rate of systemic and invasive mycoses caused by opportunistic fungal pathogens, including *Candida albicans*, *Aspergillus fumigatus*, and *Cryptococcus neoformans,* has increased over the past decades due to the extension of human lifespan and increase in the number of immunocompromised patients with organ transplantation. Recently, it has been estimated that there are more than 4,000,000 infections caused by opportunistic fungal pathogens annually [1]. Despite global concerns, therapeutic options are limited due to the evolutionarily similar cellular structures observed in both humans and fungi [2,3]. In addition, given that antifungal agents such as azole drugs, polyene drugs, and flucytosine have adverse effects [4], there is an urgent need for the identification of novel compounds and targets for treatment of this condition, for the betterment of public health.

Plants produce a large number of chemicals, some of which are used as primary metabolites, while others are used to protect themselves from pathogens and herbivores. Of the different plant defense compounds, phytoanticipins and phytoalexins have been widely characterized over several decades [5]. Phytoanticipins are pre-existing compounds found in diverse locations that are released from organelles immediately after a pathogen attack. In contrast, phytoalexins are metabolites that are synthesized de novo and accumulate following pathogen attack [6].

Terpene, a hydrocarbon compound constructed from five-carbon isoprene, is classified as a monoterpene (C10), sesquiterpene (C15), and diterpene (C20), based on the isoprene units. Terpenoids are organic chemicals derived from terpenes, with additional oxygen-containing functional group(s). Thyme oil (from *Thymus vulgaris* L., Lamiaceae) consists of diverse monoterpenoids, including thymol (10–64%), carvacrol (0.4–20.6%), and *p*-cymene (9.1–22.2%) and is used as a food additive as well as for pharmacological and cosmetic purposes [7]. Thymol, a main component of thyme oil, exhibits a variety of biological activities, such as anti-carcinogenesis and anti-inflammatory activities [8]. Thymol also exhibits antibacterial activity against an array of Gram-positive and Gram-negative strains [9,10]. In addition to its antibacterial effect, it exhibits fungicidal activity against *Candida albicans*, *Botrytis cinerea*, *Rhizopus oryzae*, and *Aspergillus* species [11,12,13,14]. Recently, Teixeira et al. analyzed the minimum inhibitory concentration and minimum fungicidal concentration values of thymol against 10 clinical strains of *C. neoformans* [15]. However, there is limited information on its mechanism of action.

*C. neoformans* is responsible for fatal meningoencephalitis in immunocompromised individuals and annually causes more than 200,000 infections globally [16]. Infectious *Cryptococcus* propagules are inhaled through the respiratory tract of the host. It then disseminates to the brain via the bloodstream and infects the central nervous system by crossing the blood–brain barrier [17,18]. Due to the availability of its genetic mutants and molecular tools, *C. neoformans* has become a model species to study fungal pathogenicity and virulence mechanisms [19]. In this study, using a reverse genetic approach, we found that thymol not only induced intracellular calcium imbalance in *C. neoformans* but also inhibited *N*-glycosylation modification of the Cpy protein. In addition, thymol treatment negatively affected ergosterol biosynthesis.

Collectively, this study provides insight into the antifungal mechanisms of *C. neoformans* modulated by natural compounds such as thymol. Furthermore, it suggests that thymol can be used as an antifungal agent or a putative supplement to currently available antibiotics, which would reduce the chance of overuse of antibiotics.

## 2. Results

### 2.1. Identification of Thymol-Responsive Signaling Pathways in C. neoformans

**Figure 1:**
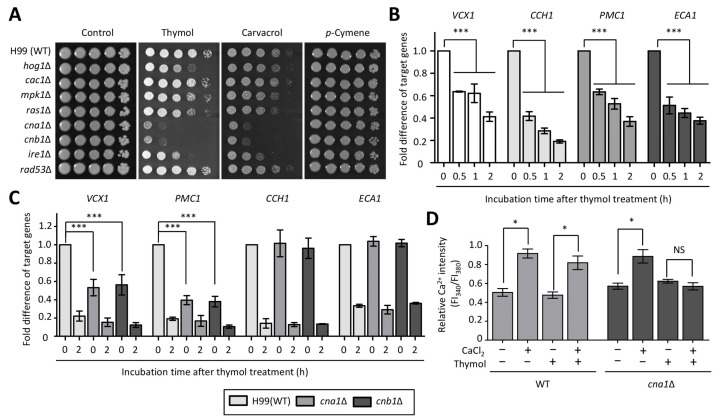
The calcineurin pathway is required for thymol resistance. (**A**) Growth inhibition of *C. neoformans* signaling mutants to monoterpenoid compounds that originate from thyme oil, including thymol, *p*-cymene, and carvacrol. (**B**,**C**) Expression levels of genes involved in calcium transport were determined using quantitative reverse transcription polymerase chain reaction (qRT-PCR) analysis with cDNA synthesized from total RNA isolated from the WT (H99) strain (**B**) and the indicated strains (**C**) treated with thymol (1 mM) during the indicated time-points. (**D**) Relative levels of intracellular Ca^2+^ concentration in the WT and *cna1*Δ mutant upon thymol treatment. The fold differences of target genes were statistically analyzed using the Bonferroni’s multiple comparison test and relative Ca^2+^ intensity was statistically analyzed using the Bonferroni’s selected comparison test performed with Prism software (* *p* < 0.05, ** *p* < 0.01, *** *p* < 0.001, and NS non-significant).

Although thyme essential oil extracted from *Thymus vulgaris* L., (Lamiaceae) is known for its antifungal activity against human fungal pathogens [11,12,15], information on alterations in signaling pathways and cellular changes within the pathogen following treatment with thyme oil remains unknown. First, we screened loss-of-function strains of *C. neoformans*, which are mutated in stress-responsive signaling pathways, for their viability post treatment with thyme oil constituents such as thymol, carvacrol, and *p*-cymene. Briefly, HOG1 encodes a high osmolarity glycerol mitogen-activated protein kinase (HOG MAPK) and is involved in the osmotic and oxidative stress response [20]. An Mpk1 kinase is a core protein of pathway for the cell wall [21]. Cna1 and Cnb1, catalytic and regulatory subunits of the calcineurin pathway, respectively, control intracellular calcium homeostasis [22]. Ire1 is a sensor kinase of unfolded protein response (UPR) and required for endoplasmic reticulum (ER) stress [23]. Cac1 (adenylyl cyclase) and Ras1 (a small GTPase) are upstream regulators of protein kinase A and are associated with growth and differentiation [24,25]. Rad53 controls DNA damage response by regulating the expression of DNA repair genes [26,27]. Spotting assays revealed that the *cna1*∆, *cnb1*∆, and *ire1*∆ mutants were sensitive to treatment with thymol and carvacrol, but not with *p*-cymene (Figure 1A). Furthermore, the *hog1*∆ mutant exhibited more sensitivity to thymol, as compared to the wild type (WT). However, the growth inhibition effects of thymol, carvacrol, and *p*-cymene on the *cac1*∆, *mpk1*∆, *ras1*∆, and *rad53*∆ mutants were negligible when compared to that on the WT (Figure 1A).

### 2.2. The Effect of Thymol Treatment on the Intracellular Ca^2+^ Balance in C. neoformans

Given the fact that carvacrol, a derivative of *p-cymene* that shows structural similarity with thymol, disrupts cellular Ca^2+^ homeostasis in *S. cerevisiae* [28] and that the relative abundance of calcium transporter genes is altered in an intracellular calcium-dependent manner [29], we determined the expression levels of the transporter genes upon thymol treatment. There was a gradual reduction in the expression levels of *VCX1* (a vacuolar Ca^2+^-exchanger), *CCH1* (a voltage-gated Ca^2+^-channel), *PMC1* (a putative vacuolar Ca^2+^-ATPase), and *ECA1* (a sarcoplasmic reticulum/ER Ca^2+^-ATPase) genes in the presence of thymol (Figure 1B). These results indicate that thymol also influences Ca^2+^ homeostasis in *C. neoformans*. These findings led us to monitor the expression levels of these genes in the *cna1*Δ and *cnb1*Δ mutants. We found that a mutation *per se* of either *CNA1* or *CNB1* resulted in reduced expression levels of *VCX1* and *PMC1*, as compared to those in the WT, at the basal level (Figure 1C), while thymol treatment resulted in no significant difference in gene expression levels between the WT and calcineurin mutants. Furthermore, the expression patterns of *CCH1* and *ECA1* in calcineurin mutants were similar to those in the WT, with or without thymol treatment (Figure 1C). Next, we measured the relative intracellular Ca^2+^ changes with FURA-2-acetoxymethyl ester (FURA-2-AM) staining post thymol treatment. We found that the relative intracellular Ca^2+^ levels in cells treated with thymol were not distinguishable from those in untreated cells (Figure 1D). Furthermore, the difference in intracellular Ca^2+^ levels was also similar between cells treated with thymol in the presence or absence of CaCl_2_. Since the expression levels of *VCX1* and *PMC1* were reduced in both the *cna1*Δ and *cnb1*Δ mutants, we compared the intracellular Ca^2+^ levels between calcineurin mutants and WT. Similar to WT, the relative intracellular Ca^2+^ levels remained unchanged in the *cna1*Δ mutant post thymol treatment. However, the relative intracellular Ca^2+^ levels in the *cna1*Δ mutant did not increase when treated with both CaCl_2_ and thymol, as compared to those in the WT. These data indicate that thymol-mediated intracellular Ca^2+^ levels are regulated in a calcineurin-dependent manner.

**Figure 2:**
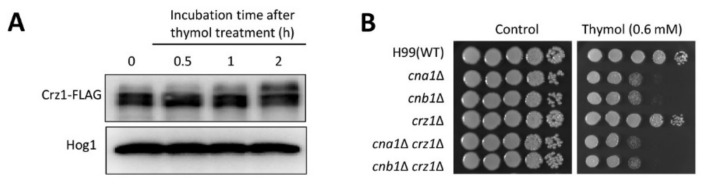
Calcineurin pathway is involved in thymol resistance in a Crz1-independent manner. (**A**) Phosphorylation of Crz1 in response to thymol. Total proteins were extracted from Crz1-FLAG strains treated with or without thymol (1 mM) for 2 h. The electrophoretic mobility of Crz1 was monitored using an anti-FLAG antibody. Anti-Hog1 polyclonal antibody was used as a loading control. (**B**) Crz1 was found to be not involved in thymol resistance in *C. neoformans*. The overnight-cultured *C. neoformans* strains were serially diluted and spotted onto a yeast-peptone-dextrose (YPD) plate containing the indicated concentration of thymol. Cells were further incubated at 30 °C and photographed daily for 3 days.

Crz1 is a well-known downstream transcription factor in the calcineurin pathway that is dephosphorylated in response to external stress [30]. To investigate the involvement of Crz1 in the mechanism of action of thymol, we monitored the dephosphorylation status of Crz1 upon thymol treatment. Crz1 was not dephosphorylated upon treatment with thymol (Figure 2A), coinciding with the observation that the *crz1*Δ mutant exhibited a similar level of thymol resistance as the WT (Figure 2B). To further demonstrate that Crz1 is dispensable for thymol resistance, we constructed *cna1*Δ *crz1*Δ and *cnb1*Δ *crz1*Δ double mutants and monitored thymol resistance using these strains. In agreement with the result of Crz1 dephosphorylation, both *cna1*Δ *crz1*Δ and *cnb1*Δ *crz1*Δ mutants were resistant to thymol, similar to the *cna1*Δ and *cnb1*Δ mutants (Figure 2B). Taken together, the calcineurin pathway modulates thymol resistance in a Crz1-independent manner.

### 2.3. The Effect of Thymol on the ER Stress in C. neoformans

**Figure 3:**
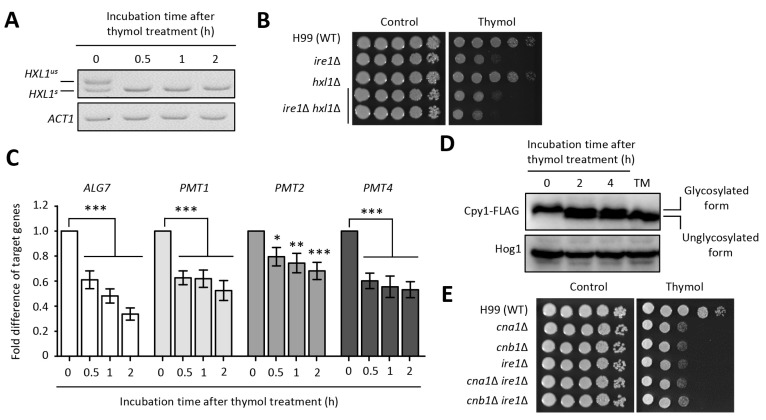
Thymol induces ER stress through reduction of protein glycosylation. (**A**) RT-PCR analysis of *HXL1* splicing upon thymol treatment. *HXL1^us^* and *HXL1^s^* indicate the unspliced and spliced versions of *HXL1*, respectively. (**B**) Ire1 controls thymol resistance in an Hxl1-independent manner. (**C**) Quantitative RT-PCR analysis of UPR downstream genes upon thymol treatment. Fold difference in the expression of the target genes was statistically analyzed using the Bonferroni’s multiple comparison test performed with Prism software (* *p* < 0.05, ** *p* < 0.01, and *** *p* < 0.001). (**D**) The glycosylation level of Cpy1 was reduced upon thymol treatment. TM is used as an inhibitor of protein glycosylation. Anti-Hog1 polyclonal antibody was used as a loading control. (**E**) The overlapping roles in thymol resistance between calcineurin and UPR pathways. TM, tunicamycin.

In addition to the calcineurin pathway, perturbation of the UPR pathway resulted in reduced growth upon thymol treatment (Figure 1A). Given that the *Cryptococcus* UPR pathway is activated by splicesome-independent splicing of *HXL1* in an Ire1-dependent manner [23], we examined whether *HXL1* splicing occurs during thymol treatment. As expected, the *HXL1* splicing event occurred post thymol treatment (Figure 3A and Figure S2A). However, the *hxl1*Δ mutant showed WT levels of thymol resistance, although deletion of Ire1, an upstream factor, rendered cells susceptible to thymol. Furthermore, the *ire1*Δ *hxl1*Δ double mutant was resistant to thymol, similar to the *ire1*Δ mutant (Figure 3B). Since the *Cryptococcus* UPR pathway regulates the expression levels of genes related to molecular chaperones (e.g., *KAR2* encoding ER-resident molecular chaperone), protein degradation (e.g., *DER1* encoding ER membrane protein involved in ER-associated degradation), vesicle-mediated transport protein (e.g., *ERV2*9 encoding proteins involved in vesicle formation), and protein glycosylation in an Hxl1-independent and -dependent manner [23], we measured the expression levels of the downstream subset in the presence of thymol. However, the expression levels of *KAR2*, *DER1*, and *ERV29* did not change in response to thymol treatment (Figure S2B). Interestingly, expression of *PMT*s (encoding *O*-mannosyltransferases involved in *O*-linked glycosylation) and *ALG7* (encoding UPD-*N*-acetyl-glucosamine-1-P transferase involved in *N*-linked glycosylation) gradually decreased with thymol treatment (Figure 3C). These results imply that thymol affects the glycan-dependent protein modification. Furthermore, we monitored a glycosylation pattern of Cpy (vacuolar carboxypeptidase Y), which undergoes *N*-glycosylation in the ER and is used as an indicator of protein glycosylation [31]. To observe glycosylation patterns using immunoblotting, we constructed strains containing Cpy (CNAG_06640) fused with a 4×FLAG epitope in the C-terminal region. Supporting the decrease in *ALG7* expression, we found that glycosylation of Cpy was reduced in the presence of thymol, indicating that thymol induces ER stress through inhibition of glycosylation (Figure 3D and Figure S2C).

To investigate the relationship between the calcineurin and UPR pathways, we constructed *cna1*∆ *ire1*∆ and *cnb1*∆ *ire1*∆ double mutants and evaluated the thymol resistance of the double mutants in comparison with that of the corresponding single mutants. Both *cna1*∆ *ire1*∆ and *cnb1*∆ *ire1*∆ exhibited similar levels of thymol resistance as the single mutants (Figure 3E), suggesting that deletion of both the calcineurin and UPR pathways does not provide additional or synergistic effects on thymol susceptibility.

### 2.4. The Effect of Thymol on the Ergosterol Biosynthesis in C. neoformans

**Figure 4:**
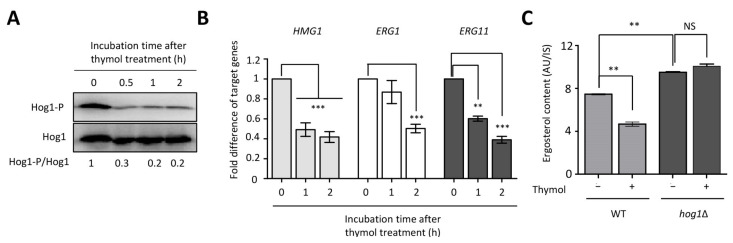
Thymol decreased cellular ergosterol content in a Hog1-dependent manner. (**A**) Thymol induced Hog1 dephosphorylation. Hog1 phosphorylation was detected using an anti-dually phosphorylated p38 antibody. Hog1 polyclonal antibody was used as a loading control. (**B**) Expression levels of gene involved in ergosterol biosynthesis reduced upon thymol treatment. cDNA was synthesized from total RNA obtained from the *C. neoformans* wild-type (H99) strain treated with or without thymol. For statistical analysis, the Bonferroni’s multiple comparison test was performed using Prism software. (**C**) Change in cellular ergosterol measurement in the WT and *hog1*Δ mutant, in the presence or absence of thymol. Relative ergosterol contents were calculated with 5α-cholestan-3β-ol as an internal control. For statistical analysis, experiments with three independent biological samples were executed. Error bars indicate S.E.M. Asterisks indicate statistical significance of difference (* *p* < 0.05, ** *p* < 0.01, *** *p* < 0.001, and NS non-significant). AU, arbitrary unit.

The finding that the *hog1*∆ mutant was sensitive to thymol treatment prompted us to test whether thymol induces Hog1 phosphorylation, because Hog1 is phosphorylated or dephosphorylated in response to external stresses in *C. neoformans* [20]. In support of the thymol-sensitivity of the *hog1*∆ mutant, Hog1 was found to be dephosphorylated in the presence of thymol (Figure 4A and Figure S3A). The fact that the viability of *Aspergillus flavus* is decreased due to reactive oxygen species (ROS) generation induced by thymol treatment and that the *Cryptococcus* HOG pathway plays a critical role in oxidative stress led us to measure intracellular ROS levels in the presence of thymol [12,20]. In contrast to *A. flavus*, thymol treatment did not produce noticeable ROS in *C. neoformans* (Figure S3B) and did not induce ROS detoxification genes (Figure S3C).

One of the major roles of the *Cryptococcus* HOG MAPK pathway is the regulation of ergosterol biosynthesis. Three genes (*HMG1* encoding HMG-CoA reductase, *ERG1* encoding squalene epoxidase, and *ERG11* encoding lanosterol 14-α demethylase) are known to be involved in the rate-limiting step of ergosterol biosynthesis [32]. We examined the expression levels of *HMG1*, *ERG1*, and *ERG11* during thymol treatment [33]. We found that the expression levels of these three genes gradually decreased upon thymol treatment (Figure 4B). To determine whether thymol inhibits ergosterol biosynthesis in a HOG MAPK pathway-dependent manner, we examined intracellular ergosterol content in the WT and *hog1*∆ mutants upon thymol treatment. Similar to a previous study [32], ergosterol content in the *hog1*Δ mutant was higher than that in the WT. In agreement with the reduced expression levels of ergosterol biosynthetic genes, ergosterol content also reduced in the WT upon thymol treatment, while it did not change in the *hog1*Δ mutant (Figure 4C). These results indicate that thymol negatively regulates ergosterol production in a HOG MAPK-dependent manner in *C. neoformans*.

**Figure 5:**
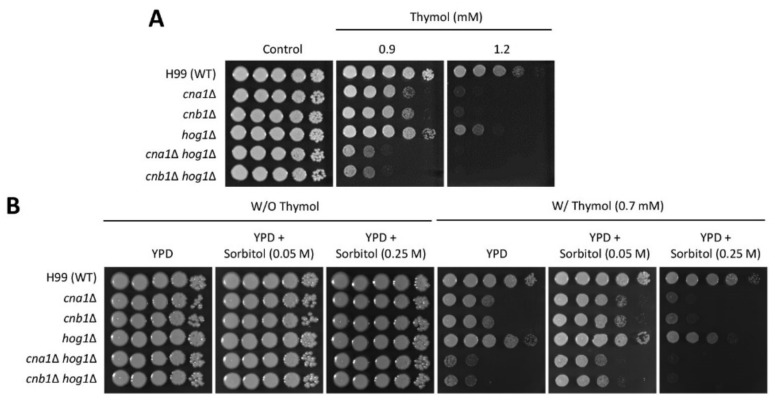
The redundant role of HOG and calcineurin pathways in thymol resistance. (**A**,**B**) Overnight-cultured cells were serially diluted (1 to 10^4^) and spotted onto YPD media containing the indicated concentration of thymol with and without sorbitol (0.05 or 0.25 M). The cells were further incubated at 30 °C for 4 days and photographed daily.

To elucidate the relationship between calcineurin and HOG pathways with respect to thymol sensitivity, we constructed *cna1*∆ *hog1*∆ and *cnb1*∆ *hog1*∆ double mutants and observed the growth of these double mutants in the presence of thymol. As expected, the *cna1*∆ *hog1*∆ and *cnb1*∆ *hog1*∆ double mutants exhibited significant growth defects compared to the corresponding single mutants in the presence of thymol (Figure 5A). This result suggests that calcineurin and HOG pathways play redundant roles in conferring thymol resistance to *C. neoformans*. Low levels of sorbitol are known to enhance membrane stability in fungal protoplasts [34], and thymol is indirectly associated with membrane damage by reducing ergosterol biosynthesis in *C. neoformans*. Therefore, we investigated whether sorbitol treatment could restore thymol-induced growth suppression in the WT and mutant strains. Overall, *C. neoformans* WT and mutant strains exhibited growth enhancement in the presence of sorbitol (0.05 M) even under thymol treatment (Figure 5B). Notably, restoration of the reduced growth upon thymol treatment was more evident in both *cna1*∆ *hog1*∆ and *cnb1*∆ *hog1*∆ mutants in the presence of sorbitol (0.05 M). However, this effect was not maintained in the presence of increased sorbitol concentration (0.25 M), which showed more severe growth defects in all of the WT and mutant strains, as compared with those without sorbitol treatment (Figure 5B). These results indicate that the susceptibility of the *hog1*∆ mutant to thymol is partly attributed to membrane instability, and that other cellular processes also cooperate in thymol resistance in a HOG MAPK-dependent manner.

## 3. Discussion

With the rapid advancement of analytical methods, novel phytochemicals have been actively isolated and functionally characterized. Recently, the antimicrobial mechanisms of action of terpenoid compounds, such as carvacrol, have been investigated in model yeasts, *S. cerevisiae* and *C. albicans*. Rao et al. demonstrated that carvacrol disturbs ion (Ca^2+^ and H^+^) homeostasis, thereby decreasing cell viability [28]. Using chemical-genetic profiling, Chaillot et al. revealed that the UPR pathway is a crucial regulator of carvacrol tolerance [35]. Transcriptome analysis performed by Ansari et al. revealed that perillyl alcohol, a monoterpene alcohol, decreases the expression levels of the calcineurin signaling pathway, and that the *cnb1*Δ homozygous mutant is sensitive to perillyl alcohol [36]. In addition to common targets, including calcineurin and UPR pathways, terpenes and terpenoids interfere with ergosterol biosynthesis and membrane integrity [36,37]. In this study, we demonstrated that thymol has conserved intracellular targets similar to those of carvacrol and perillyl alcohol in yeasts and elucidated its antifungal mechanism through a genetics approach in *C. neoformans*. First, perturbation of calcineurin and UPR signaling pathways resulted in a loss of viability in response to thymol. However, the fact that only the *ire1*Δ mutant, and not the *hxl1*Δ mutant, exhibited sensitivity to thymol in this study is in stark contrast to the previous finding that strains lacking *IRE1* or *HAC1*, which is a functional orthologous gene to *HXL1*, displayed similar resistance to carvacrol [35]. This, in turn, indicates that the *Cryptococcus* UPR pathway is evolutionarily distinct, as compared to other fungal UPR pathways. Second, many studies have reported that terpene and terpenoid compounds affect microbial cellular membranes, and thymol reduces ergosterol content, but a mechanistic view of this finding remains elusive. We found that thymol reduced ergosterol content in *C. neoformans* by modulating the expression levels of genes involved in ergosterol biosynthesis, and this regulatory mechanism was dependent on the HOG MAPK pathway.

Although terpenoids and terpenes share similar intracellular targets between fungi, regulatory mechanisms in response to these molecules are divergent. It is well known that terpene or terpenoid treatments produce ROS, which elicit oxidative stress, thereby leading to cell death. For example, thymol treatment leads to the production of ROS and nitric oxide in *A. flavus* [12]. However, in this study, we provide several lines of evidence showing that the fungicidal action of thymol in *C. neoformans* is distinct from the conserved antifungal effect caused by ROS production in other fungi. Our results show that thymol did not induce intracellular ROS production. In accordance with this result, expression levels of oxidative stress-related genes, such as *SRX1*, which is strongly induced in oxidative stress [38], were not significantly changed. The divergent effects caused by terpene or terpenoid compounds are often fungal species-specific. Geraniol treatment induces ROS accumulation in *A. flavus* but does not affect the production of ROS in *A. ochraceus* [39]. In addition to cellular responses to ROS, the regulation of intracellular Ca^2+^ levels in response to thymol is different among eukaryotes. In *S. cerevisiae*, carvacrol, thymol, and eugenol induce a rapid influx of Ca^2+^ from the extracellular medium [28]. However, we were not able to detect noticeable changes in intracellular Ca^2+^ levels in the presence of thymol in *C. neoformans*.

Intriguingly, we found that thymol affects protein glycosylation by modulating the expression of genes involved in *N*-glycosylation and *O*-glycosylation. Supporting our results, the *S. cerevisiae* strain with a deletion of *CAX4*, which encodes dolichyl pyrophosphate phosphatase in the *N*-glycosylation process, showed growth defects in response to thymol, and glycosylation-related genes were downregulated upon carvacrol treatment in *C. albicans* [35,40]. In this state, it is unclear how thymol suppresses the expression levels of glycosylation-related genes, because there was an increase in the activated form of *HXL1*, which is a positive regulator of these genes, and deletion of *HXL1* did not affect growth inhibition in response to thymol. Furthermore, it could not exclude that thymol might directly affect enzymes required for the attachment of *N*-glycan to the residues of CnCpy1. We believe that detailed mechanisms of thymol on the *N*-glycosylation process should be characterized in future studies. However, several studies have provided evidence that calcium homeostasis in the ER and Golgi is required for export of secretory proteins and protein folding. Deletion of *PMR1*, encoding high-affinity Ca^2+^/Mn^2+^ P-type ATPase, which is required for Ca^2+^/Mn^2+^ transport into the Golgi, induces defects in *N*-linked glycosylation in the presence of Ca^2+^-enriched conditions [41]. Furthermore, Colinet et al. recently reported that Gdt1, which is a Golgi-localized cation/Ca^2+^ exchanger, is required for the glycosylation of Cpy and Gas1 under high concentrations of external calcium [42]. The results of the present study that, upon treatment with thymol, there was a decrease in the expression levels of calcium transporter genes in the ER and Golgi and *N*-glycosylation of Cpy, although the relative intracellular calcium levels did not change, indicate that thymol might induce Ca^2+^ imbalance in each organelle. Therefore, the relationship between intracellular Ca^2+^ imbalance caused upon thymol treatment and *N*-linked and *O*-linked glycosylation should be of interest.

In this study, we investigated the antifungal activity of the thymol in vitro system, not in vivo system, indicating that the efficacy of the thymol in vivo system should be checked in further study. Because it has been reported that cytotoxicity induced by thymol treatment occurs in diverse cell lines [43], it would be of our interest to modify the chemical structure of thymol aiming to reduce its cytotoxic effects.

**Figure 6:**
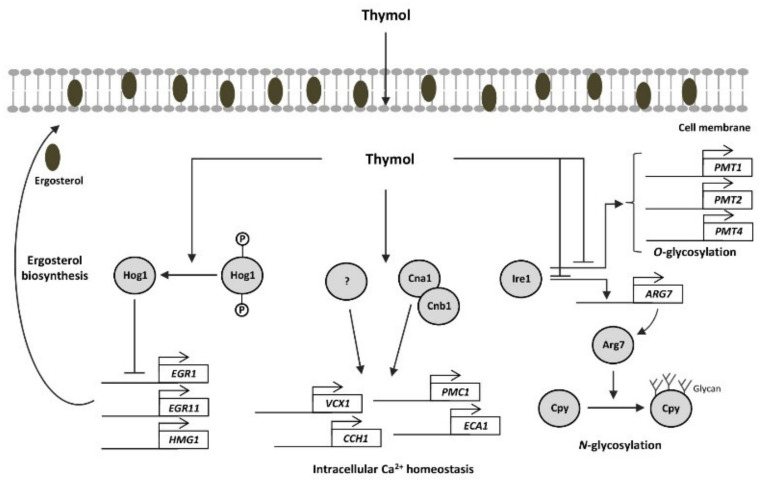
The suggested antifungal mechanism of thymol in *C. neoformans*. Thymol induced Hog1-dephosphorylation, thereby decreasing ergosterol biosynthesis. Thymol resulted in intracellular calcium imbalance by regulation of calcium transporter genes in calcineurin-dependent and -independent manners. Furthermore, thymol affects protein glycosylation by controlling expressions of genes encoding *O*-glycosylation and *N*-glycosylation.

Taken together, we suggest that thymol induces Hog1-dephosphorylation, thereby decreasing ergosterol biosynthesis by suppressing *ERG1*, *ERG11*, and *HMG1* expression. Thymol resulted in intracellular calcium imbalance by regulating calcium transporter genes in a calcineurin-dependent and -independent manner. Furthermore, thymol affects protein glycosylation by controlling the expression of genes encoding *O*-glycosylation and *N*-glycosylation (Figure 6). Therefore, this study could improve our understanding of the molecular mechanisms of the fungal pathogen in the treatment of terpene compounds.

## 4. Materials and Methods

### 4.1. Strain and Media

The strains used in this study are listed in Table S1. *C. neoformans* strains were cultured in yeast extract-peptone-dextrose (YPD) medium at 30 °C.

### 4.2. Construction of C. neoformans Mutant Strains

Deletion mutant strains were constructed in the *C. neoformans* serotype A H99S strain background using split marker/double-joint PCR strategies [19]. The genetic information for each gene was obtained from FungiDB (https://fungidb.org/fungidb/, accessed on 5 May 2018) The primers used in this study are described in Table S2. The gene deletion cassette and transformation were generated as previously described [19]. To confirm the genetic mutation of mutants, Southern blot analysis was performed using a gene-specific probe, as previously described [19].

### 4.3. Total RNA Isolation, cDNA Synthesis, and Quantitative Reverse Transcription PCR

An overnight culture of *C. neoformans* was inoculated into fresh YPD medium, such that the OD_600_ post inoculation was 0.2, and further incubated at 30 °C until the OD_600_ of the culture reached 0.8. Cells were then treated with thymol (final concentration of 1 mM) and further incubated at 30 °C for the indicated time-points. The methods for total RNA isolation, RNA purification, and cDNA synthesis have been described previously [32]. We performed quantitative reverse transcription PCR analysis with the gene-specific primers listed in Table S2 using the CFX96 Real-Time PCR Detection System (Bio-Rad, Hercules, CA, USA). Relative expression of the target genes was determined using the 2^−∆∆Ct^ method, and statistical analyses were performed using one-way analysis of variance with Bonferroni’s multiple-comparison test (GraphPad Software Inc., San Diego, CA, USA). The Actin1 gene was used as an internal control for quantification of gene expression.

### 4.4. Protein Extraction and Western Blot Analysis

The Cpy-4×FLAG culture was diluted in fresh YPD media such that the OD_600_ value post dilution was 0.2, and further incubated at 30 °C until the OD_600_ reached 0.8. Fifty milliliters of culture were pelleted for the zero-time sample. The remaining culture was treated with 1 mM thymol and further incubated for the indicated time-points. Whole-cell lysates were extracted using lysis buffer, as previously described [44]. An anti-dually phosphorylated p38 antibody (catalog number 4511, Cell Signaling Technology, Danvers, MA, USA) was used to assess the Hog1 phosphorylation level, while anti-β-actin antibody (catalog number SC-47778, Santa Cruz Biotechnology., Dallas, TX, USA) and anti-polyclonal Hog1 antibody (catalog number SC-9079, Santa Cruz Biotechnology) were used as a loading control.

### 4.5. Spotting Assay

*C. neoformans* strains were cultured in liquid YPD medium overnight at 30 °C. The cells were 10-fold serially diluted (1 to 10^4^ dilutions) in sterile distilled water and spotted onto solid YPD medium containing the indicated concentrations of terpene or terpenoid compounds. The cells were further incubated for 2–5 days and photographed daily.

### 4.6. Determination of Relative Intracellular Ca^2+^ Levels

Each *C. neoformans strain* was adjusted to 2 × 10^7^ cells/mL in 15 mL of YPD medium. Next, the cells were treated with a final concentration of 1 mM thymol, 100 nM CaCl_2_, and both thymol and CaCl_2_, and further incubated for 2 h at 30 °C. After fixation, the cells were incubated with 0.1 μM FURA-2-AM (catalog number F1201, Invitrogen, Waltham, MA, USA) for 2 h at 37 °C. After incubation, the FURA-2-AM fluorescence intensity was determined at excitation wavelengths of 340 and 380 nm and an emission wavelength of 505 nm using a fluorescence spectrophotometer (Tecan, Männedorf, Switzerland). The relative intracellular Ca^2+^ levels were expressed as the ratio of fluorescence intensity (FI) obtained by excitation at 340 and 380 nm (FI_340 nm_/FI_380 nm_).

### 4.7. Construction of Strain Containing CPY-FLAG

The strain tagged with Cpy (CNAG_06640)-4×FLAG was generated as follows: the three PCR fragments (promoter and exon of *CPY* gene (*CPY*PE), 4×FLAG tag sequence (4×FLAG), and terminator of *CPY* gene (*CPY*t)) were amplified using the primers listed in Table S2. The amplified *CPY*PE fragment was cloned into pGEM-T-Easy (catalog number A3600, Promega) to generate pGEM-*CPY*PE (KWE70). The 4×FLAG-*CPY*t fragment was generated using overlap PCR with the primers J737 and J740 and 4×FLAG and *CPY*t fragments as templates. The 4×FLAG-*CPY*t fragment was cloned into pGEM-T-Easy to construct pGEM-4xFLAG-*CPY*t (KWE71). The BamHI-digested 4×FLAG-*CPY*t fragment was subcloned into pGEM-*CPY*PE to generate the plasmid pGEM-*CPY-*4×FLAG (KWE73). Next, the NotI-digested *CPY-*4×FLAG fragment was subcloned into pJAF12 containing a *NEO* selection marker, to generate the plasmid pJAF12-*CPY-*4×FLAG (KWE81). The NsiI-digested pJAF12-*CPY-*4×FLAG was biolistically transformed into the H99S strain. The insertion of the fragment was initially screened using diagnostic PCR with the primers J737 and J740. Next, the proper production of Cpy-4×FLAG was confirmed using western blot analysis with an anti-FLAG antibody (catalog number F3165, Sigma, Saint Louis, MO, USA).

### 4.8. ROS Measurement Assay

ROS levels were measured as described previously, with minor modifications [45]. The cells were adjusted to a concentration of 1 × 10^7^ cells/mL, treated with thymol for 1 h, and then fixed with formaldehyde solution. Next, the cells were incubated with 10 µM H_2_DCFDA (catalog number D399, Invitrogen) for 2 h. After incubation, cells were washed three times with phosphate buffered saline and fluorescence was measured using fluorometry (Infinite 200) with emission and excitation wavelengths of 488 and 520 nm, respectively. Fluorescence intensity was normalized to cell density. Statistical analyses were performed using one-way analysis of variance with Bonferroni’s multiple comparison test.

### 4.9. Measurement of Ergosterol Content

The overnight culture was inoculated into fresh YPD media, such that the OD_600_ value post inoculation was 0.2, and further incubated at 30 °C until the OD_600_ reached a value of 1.0. The cell culture was then treated with thymol (final concentration, 1 mM) and further incubated for 2 h. The control cells were treated with DMSO only. After incubation for the indicated time-points, the cells were collected, frozen in liquid nitrogen, and lyophilized overnight. The dried cells were ground with a mortar and pestle. Ergosterol content was extracted as previously described, with minor modifications. Briefly, 100 mg of dried cells were homogenized with 4 mL of chloroform/methanol (2:1, *v/v*) containing 5α-cholestan-3β-ol as an internal standard. After removal of the solvent using a stream of N_2_ gas, the extract was dissolved in chloroform and then filtered using 0.2 μm hydrophobic polytetrafluoroethylene. After removal of the solvent using a stream of N_2_ gas, the resultant pellet was saponified at 90 °C for 1 h in 1 mL KOH solution (20% (*w/v*) KOH in 80% (*v/v*) ethanol). After cooling to room temperature, 1 mL of distilled water was added to each sample. Diethyl ether (2 mL) was added to the sample, and the mixture was shaken vigorously. After centrifugation, the solvent layer (supernatant) was transferred to a fresh vial. Extraction with diethyl ether was performed three times, and the collected organic solvent layer was dried with N_2_. For the derivatization step, the dried samples were dissolved in chloroform, and an equal volume of chloroform containing *N*-methyl-*N*-trimethylsilyltrifluoroacetamide was added and incubated at 80 °C for 30 min. After derivatization, the samples were dried with N_2_ and dissolved in chloroform. The dissolved samples were injected into a GC‒MS 7890A/5975C system (Agilent, Santa Clara, CA, USA) equipped with a DB-5MS capillary column (30 m × 0.25 mm ID, 0.25 μm thickness, Agilent). The analytical conditions of GC‒MS were as follows: electron impact, 70 eV; MS source temperature, 230 °C; injection temperature, 180 °C; carrier gas, helium; flow rate, 1 mL.min^−1^; column temperature program for ergosterol, 180 °C for 5 min, increased to 300 °C at 2 °C min^−1^ and held for 5 min; and ergosterol was identified in a selected ion chromatogram at *m/z* = 468 using authentic standards, as well as web-based libraries including Wiley 7th edition (https://onlinelibrary.wiley.com, accessed on 12 June 2020), and the National Institute of Standards and Technology version 2.0, (https://chemdata.nist.gov/mass-spc/ms-search, accessed on 12 June 2020). An authentic ergosterol standard (catalog number 45480, Sigma) was purchased and used for quantification.

## 5. Conclusions

The current study elucidated the antifungal mechanisms of thymol in *C. neoformans* using a reverse genetic approach. First, we found that thymol treatment affects intracellular calcium homeostasis by suppressing the expression of genes involved in the calcium transporters. Second, thymol treatment decreased expression levels of genes required for *N*-glycosylation, thereby reducing protein glycosylation. Third, we demonstrated that thymol decreased ergosterol contents in a HOG pathway-dependent manner. In conclusion, this study provides insights into the potential use of thymol as an antifungal agent and advances our understanding of the antifungal mechanisms of action of thymol.

## Data Availability

The data presented in this study are available in Supplementary information.

## Supplementary Materials

The following are available online. Figure S1: Construction of gene deletion mutants, Figure S2: Expression levels of the UPR downstream genes, Figure S3: Thymol did not induce intracellular ROS levels in *C. neoformans*, Table S1: Strains used in this study, and Table S2: Primers used in this study.

## Sample Availability

Samples of the compounds and strains are available from the authors.
