# Supplementary material for: Investigation of Antifungal Mechanisms of Thymol in the Human Fungal Pathogen, Cryptococcus neoformans
Source: Molecules. 2021 Jun 7;26(11):3476. doi: 10.3390/molecules26113476 (PMC8201179; doi:10.3390/molecules26113476)
Supplement: Supplementary file 1 [file molecules-26-03476-s001.zip › Fig_S1_Southern blot.pptx]

## Slide 1
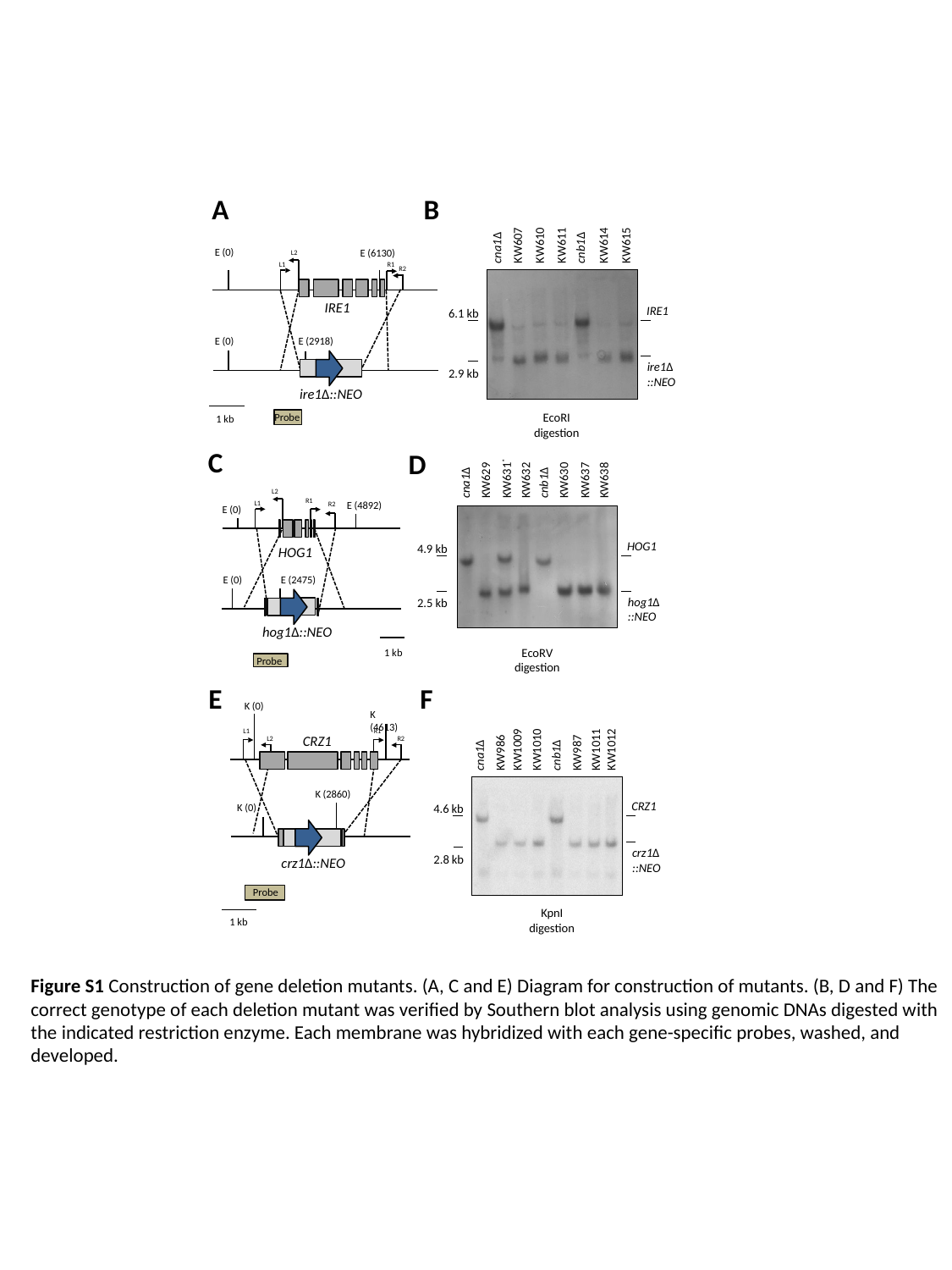

B
A
KW610
KW611
KW615
KW607
KW614
cna1Δ
cnb1Δ
E (0)
E (6130)
L2
R1
L1
R2
IRE1
IRE1
6.1 kb
E (0)
E (2918)
ire1∆
::NEO
2.9 kb
ire1∆::NEO
EcoRI
digestion
Probe
1 kb
C
D
KW631*
KW632
KW637
KW638
KW629
KW630
cna1Δ
cnb1Δ
L2
R1
E (4892)
L1
R2
E (0)
HOG1
4.9 kb
HOG1
E (2475)
E (0)
hog1∆
::NEO
2.5 kb
hog1∆::NEO
EcoRV digestion
1 kb
Probe
F
E
K (0)
K (4613)
L1
R1
CRZ1
L2
R2
KW1009
KW1010
KW1011
KW1012
KW986
KW987
cna1Δ
cnb1Δ
K (2860)
CRZ1
4.6 kb
K (0)
crz1∆
::NEO
2.8 kb
crz1∆::NEO
Probe
KpnI
digestion
1 kb
Figure S1 Construction of gene deletion mutants. (A, C and E) Diagram for construction of mutants. (B, D and F) The correct genotype of each deletion mutant was verified by Southern blot analysis using genomic DNAs digested with the indicated restriction enzyme. Each membrane was hybridized with each gene-specific probes, washed, and developed.
