# Supplementary material for: Investigation of Antifungal Mechanisms of Thymol in the Human Fungal Pathogen, Cryptococcus neoformans
Source: Molecules. 2021 Jun 7;26(11):3476. doi: 10.3390/molecules26113476 (PMC8201179; doi:10.3390/molecules26113476)
Supplement: Supplementary file 1 [file molecules-26-03476-s001.zip › Fig_S2_ER stress_revision.pptx]

## Slide 1
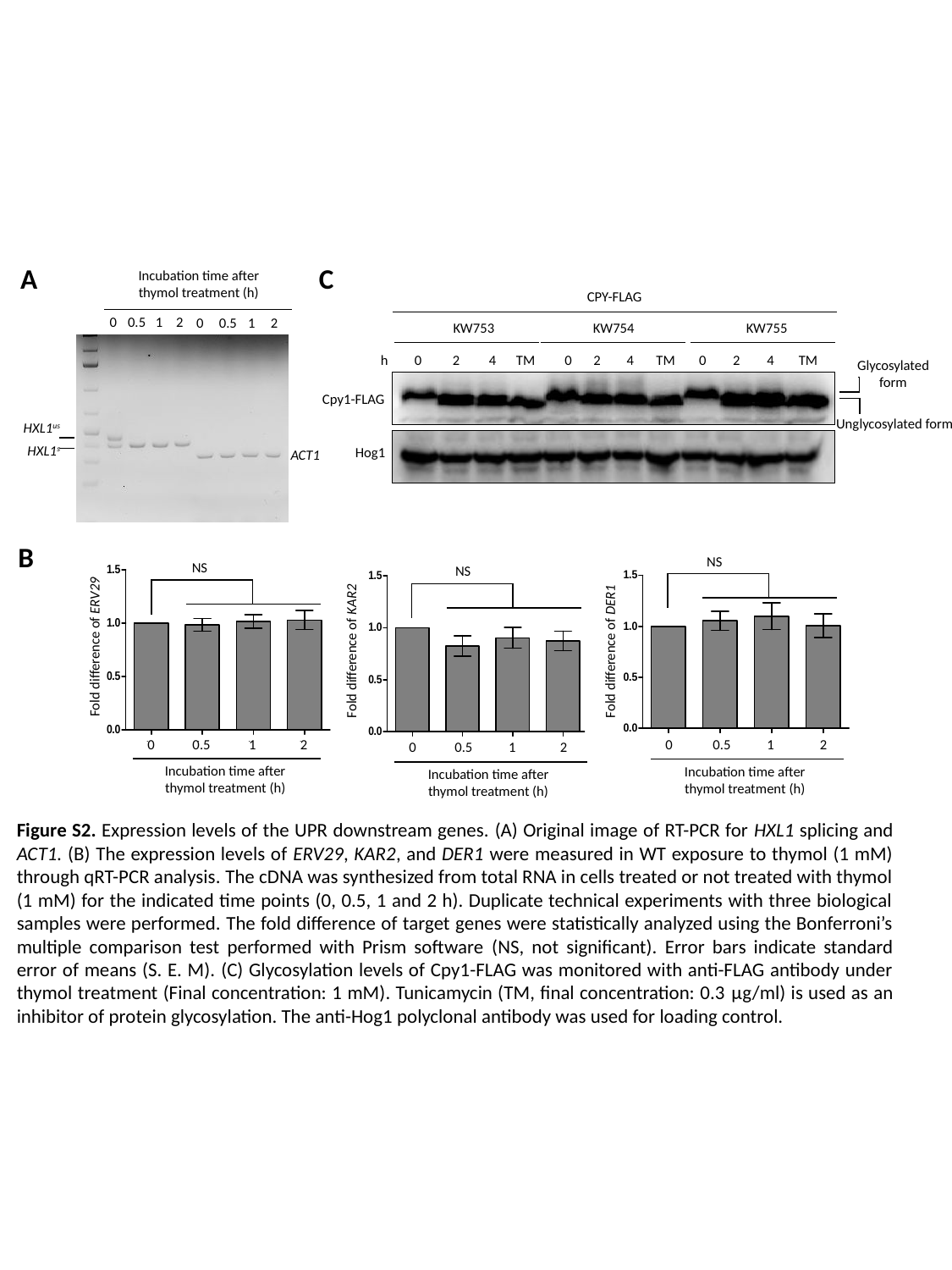

A
C
Incubation time after thymol treatment (h)
CPY-FLAG
0
0.5
1
2
0
0.5
1
2
KW753
KW754
KW755
h
0
2
4
0
2
4
TM
0
2
4
TM
TM
Glycosylated form
Cpy1-FLAG
Unglycosylated form
HXL1us
HXL1s
Hog1
ACT1
B
NS
NS
NS
Fold difference of ERV29
Fold difference of KAR2
Fold difference of DER1
0
0.5
1
2
0
0.5
1
2
0
0.5
1
2
Incubation time after thymol treatment (h)
Incubation time after thymol treatment (h)
Incubation time after thymol treatment (h)
Figure S2. Expression levels of the UPR downstream genes. (A) Original image of RT-PCR for HXL1 splicing and ACT1. (B) The expression levels of ERV29, KAR2, and DER1 were measured in WT exposure to thymol (1 mM) through qRT-PCR analysis. The cDNA was synthesized from total RNA in cells treated or not treated with thymol (1 mM) for the indicated time points (0, 0.5, 1 and 2 h). Duplicate technical experiments with three biological samples were performed. The fold difference of target genes were statistically analyzed using the Bonferroni’s multiple comparison test performed with Prism software (NS, not significant). Error bars indicate standard error of means (S. E. M). (C) Glycosylation levels of Cpy1-FLAG was monitored with anti-FLAG antibody under thymol treatment (Final concentration: 1 mM). Tunicamycin (TM, final concentration: 0.3 μg/ml) is used as an inhibitor of protein glycosylation. The anti-Hog1 polyclonal antibody was used for loading control.
