# Supplementary material for: Investigation of Antifungal Mechanisms of Thymol in the Human Fungal Pathogen, Cryptococcus neoformans
Source: Molecules. 2021 Jun 7;26(11):3476. doi: 10.3390/molecules26113476 (PMC8201179; doi:10.3390/molecules26113476)
Supplement: Supplementary file 1 [file molecules-26-03476-s001.zip › Fig_S3_ROS analysis_revision.pptx]

## Slide 1
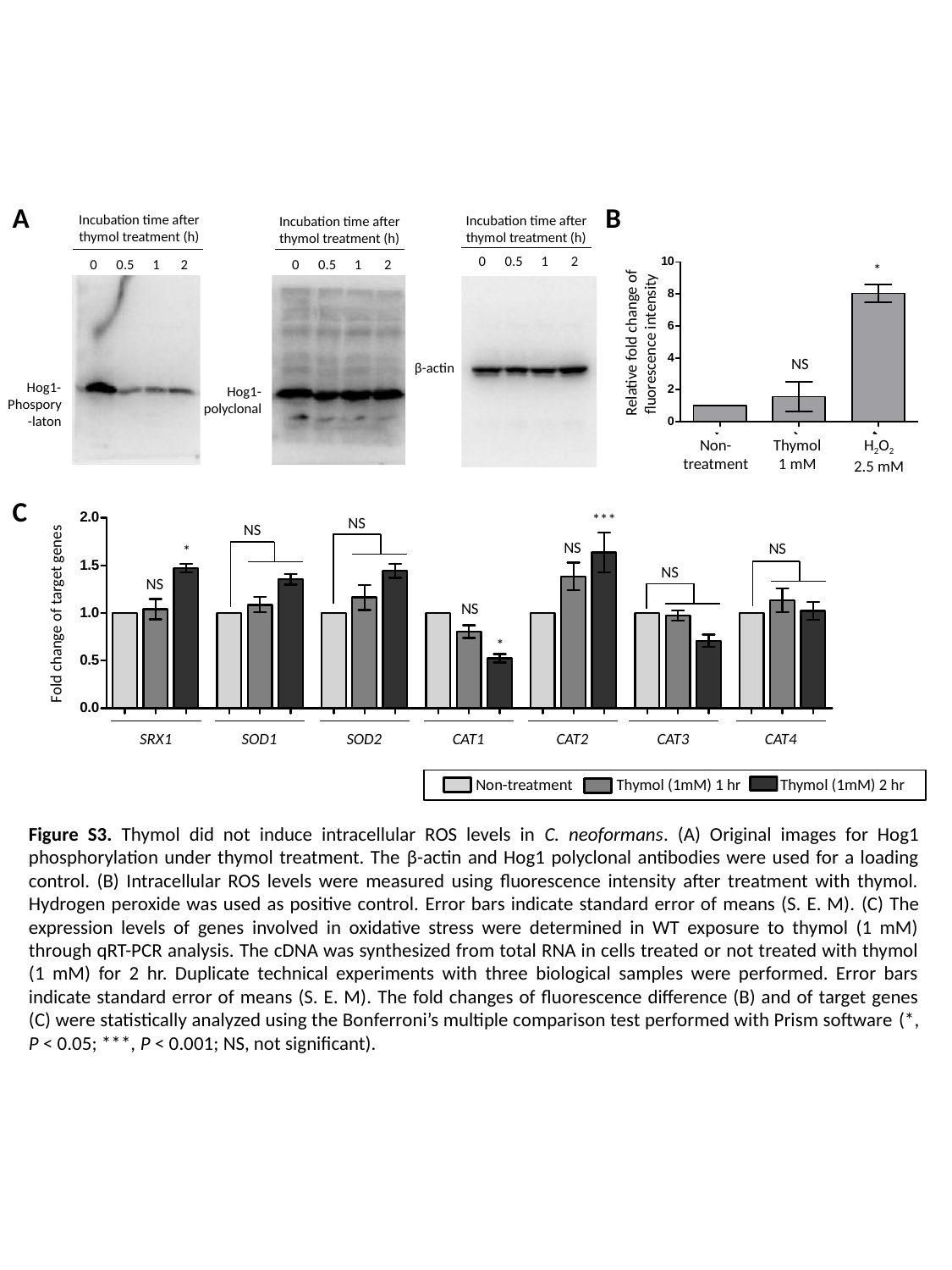

B
A
Incubation time after thymol treatment (h)
Incubation time after thymol treatment (h)
Incubation time after thymol treatment (h)
0
0.5
1
2
0
0.5
1
2
0
0.5
1
2
*
Relative fold change of fluorescence intensity
NS
β-actin
Hog1-Phospory-laton
Hog1-polyclonal
Non-treatment
Thymol
1 mM
H2O2
2.5 mM
C
***
NS
NS
NS
NS
*
NS
NS
NS
Fold change of target genes
*
SRX1
SOD1
SOD2
CAT1
CAT2
CAT3
CAT4
Non-treatment
Thymol (1mM) 1 hr
Thymol (1mM) 2 hr
Figure S3. Thymol did not induce intracellular ROS levels in C. neoformans. (A) Original images for Hog1 phosphorylation under thymol treatment. The β-actin and Hog1 polyclonal antibodies were used for a loading control. (B) Intracellular ROS levels were measured using fluorescence intensity after treatment with thymol. Hydrogen peroxide was used as positive control. Error bars indicate standard error of means (S. E. M). (C) The expression levels of genes involved in oxidative stress were determined in WT exposure to thymol (1 mM) through qRT-PCR analysis. The cDNA was synthesized from total RNA in cells treated or not treated with thymol (1 mM) for 2 hr. Duplicate technical experiments with three biological samples were performed. Error bars indicate standard error of means (S. E. M). The fold changes of fluorescence difference (B) and of target genes (C) were statistically analyzed using the Bonferroni’s multiple comparison test performed with Prism software (*, P < 0.05; ***, P < 0.001; NS, not significant).
