# Supplementary material for: Investigation of Antifungal Mechanisms of Thymol in the Human Fungal Pathogen, Cryptococcus neoformans
Source: Molecules. 2021 Jun 7;26(11):3476. doi: 10.3390/molecules26113476 (PMC8201179; doi:10.3390/molecules26113476)
Supplement: Supplementary file 1 [file molecules-26-03476-s001.zip › Table_S1_strains.pdf]

Table S1. Strains used in this study

Strain	Genotype	Parent	Reference
H99	*MATα*		[1]
YSB64	*MATα hog1Δ::NAT-STM#177*	H99	[2]
YSB42	*MATα cac1Δ::NAT-STM#159*	H99	[3]
YSB53	*MATα ras1Δ::NAT-STM#150*	H99	[3]
KK1	*MATα cna1Δ::NAT-STM#117*	H99	[4]
KK2	*MATα cnb1Δ::NAT-STM#117*	H99	[4]
KK3	*MATα mpk1Δ::NAT-STM#150*	H99	[4]
YSB552	*MATα ire1Δ::NAT-STM#224*	H99	[5]
YSB723	*MATα hxl1Δ::NAT-STM#295*	H99	[5]
YSB3785	*MATα rad53Δ::NAT-STM#184*	H99	[6]
YSB1263	*MATα crz1Δ::NAT-STM#231*	H99	[7]
KW308	*MATα ire1Δ::NAT-STM#224 hxl1Δ::NEO*	YSB552	[8]
KW309	*MATα ire1Δ::NAT-STM#224 hxl1Δ::NEO*	YSB552	[8]
KW610	*MATα cna1Δ::NAT-STM#117 ire1Δ::NEO*	KK1	This study
KW611	*MATα cna1Δ::NAT-STM#117 ire1Δ::NEO*	KK1	This study
KW614	*MATα cnb1Δ::NAT-STM#117 ire1Δ::NEO*	KK2	This study
KW615	*MATα cnb1Δ::NAT-STM#117 ire1Δ::NEO*	KK2	This study
KW629	*MATα cna1Δ::NAT-STM#117 hog1Δ::NEO*	KK1	This study
KW632	*MATα cna1Δ::NAT-STM#117 hog1Δ::NEO*	KK1	This study
KW630	*MATα cnb1Δ::NAT-STM#117 hog1Δ::NEO*	KK2	This study
KW637	*MATα cnb1Δ::NAT-STM#117 hog1Δ::NEO*	KK2	This study
KW638	*MATα cnb1Δ::NAT-STM#117 hog1Δ::NEO*	KK2	This study
KW986	*MATα cna1Δ::NAT-STM#117 crz1Δ::NEO*	KK1	This study
KW1009	*MATα cna1Δ::NAT-STM#117 crz1Δ::NEO*	KK1	This study
KW1010	*MATα cna1Δ::NAT-STM#117 crz1Δ::NEO*	KK1	This study
KW987	*MATα cnb1Δ::NAT-STM#117 crz1Δ::NEO*	KK2	This study
KW1011	*MATα cnb1Δ::NAT-STM#117 crz1Δ::NEO*	KK2	This study
KW1012	*MATα cnb1Δ::NAT-STM#117 crz1Δ::NEO*	KK2	This study
KW753	*MATα* CPY-4xFLAG-NEO	H99	This study
KW754	*MATα* CPY-4xFLAG-NEO	H99	This study
KW755	*MATα* CPY-4xFLAG-NEO	H99	This study

Each *NAT-STM#* indicates the Nat^r marker with a unique signature tag.

References

[1] J.R. Perfect, N. Ketabchi, G.M. Cox, C.W. Ingram, C.L. Beiser, Karyotyping of *Cryptococcus neoformans* as an epidemiological tool, J. Clin. Microbiol. 31 (1993) 3305-3309.

[2] Y.S. Bahn, K. Kojima, G.M. Cox, J. Heitman, Specialization of the HOG pathway and its impact on differentiation and virulence of *Cryptococcus neoformans*, Mol Biol Cell 16 (2005) 2285-2300.

[3] Y.S. Bahn, J.K. Hicks, S.S. Giles, G.M. Cox, J. Heitman, Adenylyl cyclase-associated protein Aca1 regulates virulence and differentiation of *Cryptococcus neoformans* via the cyclic AMP-protein kinase A cascade, Eukaryot Cell 3 (2004) 1476-1491.

[4] K. Kojima, Y.S. Bahn, J. Heitman, Calcineurin, Mpk1 and Hog1 MAPK pathways independently control fludioxonil antifungal sensitivity in *Cryptococcus neoformans*, Microbiology 152 (2006) 591-604.

[5] S.A. Cheon, K.W. Jung, Y.L. Chen, J. Heitman, Y.S. Bahn, H.A. Kang, Unique evolution of the UPR pathway with a novel bZIP transcription factor, Hxl1, for controlling pathogenicity of *Cryptococcus neoformans*, PLoS Pathog. 7 (2011) e1002177.

[6] K.T. Lee, Y.S. So, D.H. Yang, K.W. Jung, J. Choi, D.G. Lee, H. Kwon, J. Jang, L.L. Wang, S. Cha, G.L. Meyers, E. Jeong, J.H. Jin, Y. Lee, J. Hong, S. Bang, J.H. Ji, G. Park, H.J. Byun, S. Park, Y.M. Park, G. Adedoyin, T. Kim, A.K. Averette, J.S. Choi, J. Heitman, E. Cheong, Y.H. Lee, Y.S. Bahn, Systematic fungal analysis of kinases in the fungal pathogen *Cryptococcus neoformans*, Nat Commun 7 (2016) 12766.

[7] K.W. Jung, D.H. Yang, S. Maeng, K.T. Lee, Y.S. So, J. Hong, J. Choi, H.J. Byun, H. Kim, S. Bang, M.H. Song, J.W. Lee, M.S. Kim, S.Y. Kim, J.H. Ji, G. Park, H. Kwon, S. Cha, G.L. Meyers, L.L. Wang, J. Jang, G. Janbon, G. Adedoyin, T. Kim, A.K. Averette, J. Heitman, E. Cheong, Y.H. Lee, Y.W. Lee, Y.S. Bahn, Systematic functional profiling of transcription factor networks in *Cryptococcus neoformans*, Nat Commun 6 (2015) 6757.

[8] K.W. Jung, Y.S. So, Y.S. Bahn, Unique roles of the unfolded protein response pathway in fungal development and differentiation, Sci Rep 6 (2016) 33413.
