# Supplementary material for: Investigation of Antifungal Mechanisms of Thymol in the Human Fungal Pathogen, Cryptococcus neoformans
Source: Molecules. 2021 Jun 7;26(11):3476. doi: 10.3390/molecules26113476 (PMC8201179; doi:10.3390/molecules26113476)
Supplement: Supplementary file 1 [file molecules-26-03476-s001.zip › Table_S2_primers.pdf]

Table S2. Primers used in this study

Primer Name	Sequence (5’- -3’)	Comment
B79	TGTGGATGCTGGCGGAGGATA	Screening primer on *ACT1* promoter
J12579	TTCCCACCCTCAGCAACGCC	Screening primer on *TRP1* terminator
B1026	GTAAAACGACGGCCAGTGAGC	M13 forward (extended)
B1027	CAGGAAACAGCTATGACCATG	M13 reverse (extended)
B1886	TGGAAGAGATGGATGTGC	NEO-NSL2
B1887	ATTGTCTGTTGTGCCCAG	NEO-NSR2
B1648	AGGAATACGAGGTTTATCGG	*IRE1* – 5’ screening primer
B1644	GCCCCATCATCATAATCAC	*IRE1* – left flanking primer 1
B1645	GCTCACTGGCCGTCGTTTTACACTATGTGTCCATCTGAGGC	*IRE1* – left flanking primer 2
B1646	CATGGTCATAGCTGTTTCCTGAGTGAGTTGAGGGAGGAAAG	*IRE1* – right flanking primer 1
B1647	GAAGAAGAGCGTCAAGAAGG	*IRE1* – right flanking primer 2
B1683	AGCATTAGGGGTGTAGGTG	*IRE1* – probe primer 1 for Southern blot
J844	AAATCGTCCGTGAAGTCGCAGG	*HOG1* – 3’ screening primer
J839	TGTGGTAGGTGCGTTATCG	*HOG1* – left flanking primer 1
J840	TCACTGGCCGTCGTTTTACAGAAAGCCCATCCATCAG	*HOG1* – left flanking primer 2
J841	CATGGTCATAGCTGTTTCCTGTCTTGGTAAGTCTCTGTGCC	*HOG1* – right flanking primer 1
J842	TACTCAACCCCATACTCACTCCCG	*HOG1* – right flanking primer 2
J845	AATCAAACACCTCGGCGGCAAC	*HOG1* – probe primer 1 for Southern blot
B5251	CACTCCATTCCTTTCTGC	RT-PCR primer L1 for *HXL1*
B5252	CGTAACTCCACTGTGTCC	RT-PCR primer R1 for *HXL1*
C51	CTTCCAGCCTTCTCTCCTTG	RT-PCR primer L1 for *ACT1*
C52	AGAGGTCCTTCCTGATGTCG	RT-PCR primer R1 for *ACT1*
C53	CTCTGAGGACGACAAGGACA	qRT primer 1 for *KAR2*
C54	AGCTCAGAAAGCTGCTCCTC	qRT primer 2 for *KAR2*
J846	GTCGCTATCCAGGGACATTT	qRT primer 1 for *ALG7*
J847	GTCCAAAGAGTTGAGGGCAT	qRT primer 2 for *ALG7*
J848	GCATTGGTTACGGTCTCCTT	qRT primer 1 for *ERV29*
J849	GCAAAGAGTCGGAGAGAACC	qRT primer 2 for *ERV29*
J850	CTCGTACGCTGTGCTTGATT	qRT primer 1 for *PMT1*
J851	CCGAGCTCATAAACCCATCT	qRT primer 2 for *PMT1*
J852	TTCCTTCACCTTGGTCTTCG	qRT primer 1 for *PMT2*
J853	CAAGCTGACAGTTGAGAACC	qRT primer 2 for *PMT2*
J854	ACAATGCGGGTCTTACTTCC	qRT primer 1 for *PMT4*
J855	TGCTGACCCTCATTACGTGT	qRT primer 2 for *PMT4*
J61	TTCCCGCCTCACTTCAATC	qRT primer 1 for *ERG1*
J62	AGGAAGACCCTGGATGGAG	qRT primer 2 for *ERG1*
J1340	ACGGTGTTGAAGACGACAG	qRT primer 1 for *HMG1*
J1341	CCGCGTGAGCGTTAAATC	qRT primer 2 for *HMG1*
B677	AATCTCCTTACCAGCCATTCGG	qRT primer 1 for *ERG11*
B678	TTCAGGGAACTTGGGAACAGC	qRT primer 2 for *ERG11*
J732	GCGGCCGCAAGGAAAGGAAACAGAACT	Primer 1 for *CPYPE* (CNAG_06640)
J733	ggatccCTCGTCTTCAGCAGCAGCC	Primer 2 for *CPYPE* (CNAG_06640)
J734	ACTTACCGCCTTCTTCGTC	Primer 1 for *CPYPE* sequencing
J735	CTTGCTCTCTCTCACCCTG	Primer 2 for *CPYPE* sequencing
J736	CCAACATTGCCAGCGTAC	Primer 3 for *CPYPE* sequencing

J875	GACAAGTCTCCCGAGAAGG	Primer 4 for *CPY*PE sequencing
J737	ggatccGGCGCGCCAGATTACAAGGA	Primer 1 for 4xFLAG tagging
J738	TTAGTCGACCTTATCGTCGTC	Primer 2 for 4xFLAG tagging
J739	GACGACGATAAGGTCGACTAATGGTAACTTTGTCGATATTT	Primer 1 for *CPY*t (CNAG_06640)
J740	ggatccGCGGCCGCAGAAGAAGGCGTACGGCTCG	Primer 2 for *CPY*t (CNAG_06640)
J399	ACCAACGGCTGTACCTCTG	qRT primer 1 for *SOD1*
J400	ATATTACCGAGGTCACCAACG	qRT primer 2 for *SOD1*
J401	CAAGGCCACCAAGAAGCTC	qRT primer 1 for *SOD2*
J402	TAGAAAGCGTGCTCCCAG	qRT primer 2 for *SOD2*
J403	TTATTCACGCGGTAAGGGAG	qRT primer 1 for *CAT1*
J404	GAAATCTCCAGGCAAGCAAG	qRT primer 2 for *CAT1*
J405	ACCGAGACAACGGATGAGG	qRT primer 1 for *CAT2*
J406	TGACACGAGACATTGGTGATC	qRT primer 2 for *CAT2*
J407	TGCCTTCGTATCCAAGCTC	qRT primer 1 for *CAT3*
J408	ATTGCGTAGCATTGCCGAC	qRT primer 2 for *CAT3*
J409	ATCGCCTGATCTTGGAGC	qRT primer 1 for *CAT4*
J410	CGCCAAAGCCATTATGAGTAC	qRT primer 1 for *CAT4*
J389	GCCACTCAAGACTGATCCTAC	qRT primer 1 for *SRX1*
J390	CGTTTCCCATCCCAGTCTC	qRT primer 1 for *SRX1*
B679	CGCCCTTGCTCCTTCTTCTATG	qRT primer 1 for *ACT1*
B680	GACTCGTCGTATTCGCTCTTCG	qRT primer 2 for *ACT1*
J1178	CGTTGGTAATGCAGCCGAG	qRT primer 1 for *VCX1*
J1179	AAGGCGATTTGGATCGAGG	qRT primer 2 for *VCX1*
J1180	CCATGCTGATGATGCTTGC	qRT primer 1 for *CCH1*
J1181	TGTGACTCTTTCGGTCTTGG	qRT primer 2 for *CCH1*
J1182	GCTTGGTGGTCGTGATTG	qRT primer 1 for *PMC1*
J1183	CAAGAAGTCGGGCAAATGG	qRT primer 2 for *PMC1*
J1184	CCGTCGAGGAGGGTAAATC	qRT primer 1 for *PMR1*
J1185	GGTTGAGAGGGTGATGAGG	qRT primer 2 for *PMR1*
J1186	GGCTGTGCAACTGTATTTGG	qRT primer 1 for *ECA1*
J1187	ACAGAGGAGCATTGGTGG	qRT primer 2 for *ECA1*
